# Assessment of cardiovascular risk factors in people with HIV infection treated with ART in rural South Africa: a cross sectional study

**DOI:** 10.1186/s12981-015-0083-6

**Published:** 2015-12-10

**Authors:** Felistas Mashinya, Marianne Alberts, Jean-Pierre Van geertruyden, Robert Colebunders

**Affiliations:** Department of Medical Science, Public Health and Health Promotion, School of Health Care Sciences, Faculty of Health Sciences, University of Limpopo, Sovenga, P. Bag X1106, Polokwane, South Africa; International Health Unit, Department of Epidemiology and Social Medicine, Faculty of Medicine, Antwerp University, Universiteitsplein 1, 2610 Antwerpen, Wilrijk, Belgium

**Keywords:** Cardiovascular disease risk, Human immunodeficiency virus, Antiretroviral therapy

## Abstract

**Background:**

The risk of cardiovascular diseases (CVD) in human immunodeficiency virus (HIV) infected people on antiretroviral therapy (ART) from some rural parts of Africa is not well known. We assessed CVD risk factors, the estimated 5-year Data collection on adverse effects of anti-HIV drugs (DA.) risk score and the 10-year Framingham risk score in persons with HIV infection on ART in a rural area in South Africa.

**Methods:**

A cross-sectional study in which the data on demographic, lifestyle, and chronic disease were collected using the World Health Organization Stepwise approach to surveillance questionnaire. Biochemical parameters were tested using standard biochemical methods. CD4 counts were performed using PIMA analyser and viral load was tested using the branched deoxyribonucleic acid technique. Student *t* test and Chi square test were used on continuous and categorical variables respectively. Bivariate and multivariate logistic regression were used to analyze predictors of CVD risk factors. Estimates of 5 and 10-year CVD risk were calculated using online tools. The Cohen’s kappa coefficient was used to assess the agreement between CVD risk equations.

**Results:**

The mean age of participants was 44.8 ± 11.8 years; 79.9 % were females. Most of the participants (85 %) had an undetectable viral load and a mean CD4 count of 462 ± 235 cell/mm^3^. The most common CVD risk factors were low high density lipoprotein cholesterol (HDL-C) (43.8 %), hypercholesterolaemia (33.2 %) and a high Apolipoprotein (Apo) B/ApoA ratio (45.4 %).Using the Framingham equation, 6.7 % of participants had a moderate to high 10-year CVD risk while the DAD risk equation showed that 31.1 % of participants had a moderate to high 5-year CVD risk. Most participants had a low CVD risk by both risk equations. The level of agreement between the two risk equations was 73.8 % (k = 0.23; 95 % CI 0.10–0.35; p value 0.001).

**Conclusion:**

CVD risk factors were common among this rural population on ART. The high proportion of participants with a moderate to high CVD risk, observed with the DAD risk equation, clearly represents a considerable health burden that can possibly be reduced by increasing educational programs on CVD prevention for people on ART. There is however a need to develop and evaluate a race/ethnicity-specific CVD risk estimation tool for HIV infected Africans.

## Background

Human immunodeficiency virus (HIV) or antiretroviral therapy (ART) through direct or indirect mechanisms may induce diabetes mellitus, dyslipidaemia, hypertension, lipodystrophy and endothelial dysfunction [1]. Furthermore, some studies have shown that traditional risk factors for cardiovascular diseases (CVD) such as low physical activity, increased body mass index (BMI), smoking, are frequently present in HIV infected people [2, 3].

A limited number of studies have assessed CVD risk factors in HIV infected people on ART from low-income countries [4–6]. Furthermore, the number of CVD risk factors, type and duration of ART reported in these studies varied. Most studies on CVD risk factors in persons with HIV infection were done in Western countries [7–11]. While some of these studies were conducted in areas with people of a low-socio-economic class similar to developing countries, differences in ethnicity, environmental, genetic factors, [12–14] and populations’ uptake of smoking cessation campaign [9], may have influenced prevalence rates of CVD risk factors.

Studies on CVD risk factors among HIV infected people are however scarce in South Africa [15, 16]. A recent attempt to describe the CVD risk in rural people on ART from Mpumalanga in South Africa was made [17], but the use of ART was self-reported and according to the authors, the prevalence of CVD risk factors in that rural population on ART may be underestimated since there remains considerable stigma associated with HIV. In South Africa, the intersection of an epidemiological transition [18], a high number of people living with HIV [19] and a widespread adult treatment coverage that was close to 80 % by mid-2011 [20], presents a high risk of CVD among people infected with HIV and needs to be investigated. While CVDs are preventable, little is known regarding CVD risk factors in HIV infected rural South Africans on ART [17]. We determined the prevalence of CVD risk factors, the estimated 5-year DAD risk score and the 10-year Framingham risk score in persons with HIV infection on ART in a rural area in South Africa.

## Methods

### Study design

A cross-sectional study was conducted in the primary health care clinics, Seobi-Dikgale, Sebayeng and Dikgale. At least 74 people with HIV infection are treated at Seobi-Dikgale clinic, while 373 people and 377 people with HIV infection are treated at Sebayeng and Dikgale clinics respectively. The three clinics are situated within the Dikgale Health and Demographic Surveillance System (HDSS) site. Dikgale HDSS site is situated about 70 km to the Northeast of Polokwane, the capital city of Limpopo Province. The site consists of 15 villages. Dwellings in Dikgale HDSS are a mix of shacks, conventional brick houses and traditional mud huts in the Limpopo province of South Africa.

### Study participants

During one month all patients, at least 15 years old who came to collect their ART were asked to participate in the study. Those willing to participate, were advised on the participation dates and time (7:00am) at their clinic and were advised to provide a fasting sample. Participants who could not come on the scheduled date were visited in their homes on an arranged date and participated. Participants were only included after written informed consent was obtained through the completion of a consent form approved by the Medical University of South Africa (MEDUNSA) Ethics committee. In the case of minors, written informed consent was obtained from their legal guardians. Pregnant women were excluded.

### Ethical considerations

Ethical approval was obtained from the Ethics Committee of University of Limpopo, Medunsa campus. Permission to conduct the study in the Dikgale HDSS site clinics was obtained from the Department of Health-Provincial office and Primary Health Care Capricorn District office.

### Data collection

The study was performed from September 2013 to March 2014. The World Health Organization stepwise approach to surveillance (WHO STEPS) questionnaire [21] was used to obtain information on dietary intake, physical activity, socio-demographic, tobacco use, alcohol consumption and medical history.

### Anthropometric and blood pressure measurements

Anthropometric and blood pressure measurements were taken following procedures as previously described [22]. In brief, weight was measured to the nearest 0.1 kg, using Omron BF 400 (Omron Healthcare, Japan). Height was measure to the nearest 0.1 cm, using a stadiometer. BMI was calculated as weight (kg)/height (m^2^). A BMI was considered as normal (18.50–24.99), overweight (25.00–29.99) and obese (≥30.00) [23]. Waist circumference and hip circumference were measured to the nearest 0.1 cm, using a measuring tape. Both parameters were used to calculate the waist to hip ratio. The Omron M5-1 (Omron Healthcare, Kyoto, Japan) was used to measure blood pressure. High blood pressure was defined as a systolic blood pressure (SBP) above 140 mmHg and/or a diastolic blood pressure (DBP) above 90 mmHg [24] and/or self-reported history of antihypertensive medication. Metabolic syndrome was defined as any three of the following five risk factors; abdominal obesity (waist circumference → 88 cm for females and >102 cm for males), high TG concentration (≥1.7 mmol/l), low HDL-C concentration (≤1.3 for females and ≤1.1 for males), high Blood pressure and raised fasting plasma glucose concentration (>7 mmol/l) [25].

### Blood collection

Fasting venous blood samples were drawn by registered nurses. Whole blood was used to measure CD4 counts on the day of collection. Serum from clotted blood and plasma from whole blood were separated through centrifugation at 2000 rpm for 15 min. Glucose was analysed soon after centrifugation using plasma from sodium fluoride tubes. The remaining samples were stored at −80 °C until analysis.

### Biochemical analysis

Triglycerides (TGs), total cholesterol (TC), high density lipoprotein (HDL) cholesterol and glucose levels were determined on ILab 300 Plus Chemistry System (Instrumentation Laboratory Company, Milan, Italy). Apolipoprotein B (ApoB), Apolipoprotein A1 (ApoA1) were measured on the IMMAGE Immunochemistry System (Beckman Coulter, USA). CD4 count was measured using the Pima Analyser (Inverness Medical, Tokyo, Japan). Viral load testing, using the branched deoxyribonucleic acid (DNA) technique (Siemens, South Africa) was performed by Toga Molecular Biology and Pathology medical laboratory that is South African National Accreditation System (SANAS) accredited for ISO 17025.

### Statistical analysis

Statistical analysis was performed using Statistical Package for Social Science version 22. Frequency histogram and line graph was used to check normality of continuous variable. The independent Student t-test was used to compare continuous variables and the Chi square test was used to compare categorical variables between males and females. Factors significant at p-value less or equal to 0.25 in bivariate regression were considered as candidates for multivariate modelling. Multivariate logistic regression was used to determine the significant predictors of CVD risk factors. The level of significance for statistical analysis was set at less than 0.05. Framingham 10-year CVD risk estimation was calculated for each participant above 20 years, with no diabetes and no history of CVD, by entering the following variables: age, gender, TC, HDL-C, SBP, smoking status and current treatment for high blood pressure, as required by the Framingham risk model tool [26]. Participants were regarded as low risk, moderate risk, or high risk when the risk score for developing CVD in 10 years was <10, 10–20 or >20 % respectively [27]. Variables included in the 5-year DAD risk estimation tool were age, sex, SBP, TC, HDL-C, diabetes mellitus, smoking status, family history of CVD, current use of abacavir, indinavir, or lopinavir and duration on indinavir and lopinavir [28]. The risk of developing coronary heart disease in the next 5-years was regarded as low (<1 %), moderate (1–5 %), high (5–10 %), or very high (>10 %) [29]. The level of agreement between DAD and Framingham risk equations was determined using Cohen’s Kappa coefficient with 95 % CI. For comparison with Framingham, participants with high and very high scores according to the DAD equation were combined and considered as high risk group. Kappa coefficients was interpreted as poor agreement (<0), slight agreement (0.0–0.20), fair agreement (0.21–0.40), moderate agreement (0.41–0.60), substantial agreement (0.61–0.80) and perfect agreement (0.81–1.00) [30].

## Results

### Characteristics of participants

Two hundred and fourteen HIV infected people on ART, participated in the study. Of which 171 (79.9 %) were females and 43 (20.1 %) were males. The mean age of ART participants was 44.8 ± 11.8 years and males were significantly older than females (49.9 ± 11.1 vs 43.5 ± 11.6, p = 0.001). The percentage of unmarried participants was 164 (76.6 %) with more females than males (80.1 vs 62.8 %, p < 0.05). About 45.8 % had secondary level of education. Unemployment was 69.6 %, and higher in females (75.4 %) than in males (46.5 %).

The majority of participants were on efavirenz (86 %) and nevirapine (12.5 %) based ART. Only three of the participants (1.5 %) were on a lopinavir/ritonavir based ART regimen. The mean duration of ART in this study was 36 months (range 1–121 months). Most of the participants (85 %) had an undetectable viral load and a mean CD4 count of 462 ± 235 cell/mm^3^.

### Prevalence of behavioural and metabolic CVD risk factors

Tobacco and alcohol use were higher among males than females respectively (39.5 vs 16.5 %, p = 0.001) and (41.9 vs 17.0 %, p = 0.001), while more females than males were physically inactive (29.1 vs 3.3 %, p = 0.002). Hypertriglyceridaemia (35.0 vs 12.5 %, p = 0.001), high TG/HDL-C ratio (37.5 vs 13.7 %, p = 0.002) and high TC/HDL ratio (27.5 vs 10.1 %, p = 0.01) was present more often in males than in females (Table 1). None of participants indicated a family history of cardiovascular disease.

**Table 1:** Demographics, HIV information and CVD risk factors in ART treated HIV infected participants by gender

	All participants N = 214, n (%)	Females N = 171, n (%)	Males N = 43, n (%)	P-value
Demographics				
Age (years)	44.8 ± 11.8	43.5 ± 11.6	49.9 ± 11.1	*0.001*
Unmarried	164 (76.6)	137 (80.1)	27 (62.8)	*0.03*
Secondary education	98 (45.8)	83 (48.5)	15 (34.9)	0.12
Unemployed	149 (69.6)	129 (75.4)	20 (46.5)	0.001
HIV information				
CD4 count (cells/mm^3^)	461.9 ± 235.3	485.5 ± 234.1	364.3 ± 216.6	*0.001*
Viral load (copies/ml)	≤50	≤50	≤50	0.14
Mean duration of ART in months (N = 200)	36.1 ± 24.4	37.0 ± 24.3	32.5 ± 24.6	0.31
NVP (NNRTI)-based ART*	25 (12.5)	22 (13.8)	3 (7.5)	
EFV (NNRTI)-based ART	172 (86)	136 (85.0)	36 (90.0)	
Alluvia (PI)-based ART	3 (1.5)	2 (1.3)	1 (2.5)	
CVD risk factors				
Tobacco use	45 (21.1)	28 (16.5)	17 (39.5)	*0.001*
	47 (22.0)	29 (17 %)	18 (41.9 %)	*0.001*
Alcohol use Physical activity <600 MET-min/wk)	38 (24.2)	37 (29.1 %)	1 (3.3 %)	*0.002*
Fruit and vegetables intake (<5 servings/day)	150 (95.5)	121 (95.3)	29 (96.7)	1.00
Obesity	27 (12.7)	25 (14.6)	2 (4.8)	0.12
Abdominal obesity	51 (23.9)	44 (25.9)	7 (16.3)	0.23
Hypertension	56 (26.2)	40 (23.4)	16 (37.2)	0.08
Diabetes	10 (4.7)	6 (3.5)	4 (9.3)	0.12
Low HDL	91 (43.8)	71 (42.3)	20 (50.0)	0.38
Hypercholesterolaemia	69 (33.2)	58 (34.5)	11 (27.5)	0.46
Hypertriglyceridaemia	35 (16.8)	21 (12.5)	14 (35.0)	*0.001*
TC/HDL (≥5)	28 (13.5)	17 (10.1)	11 (27.5)	*0.01*
High TG/HDL-C (≥1.49)	38 (18.3)	23 (13.7)	15 (37.5)	*0.001*
ApoB/ApoA (≥0.68)	93 (45.4)	74 (44.8)	19 (47.5)	0.86
Metabolic syndrome	20 (9.6)	15 (8.9)	5 (12.5)	0.56

Diabetes—glucose >7 mmol/l and/or history of diabetes, hypertension—systolic blood pressure >140 and/or diastolic blood pressure >90 or history of high blood pressure, abdominal obesity—waist circumference >88 cm for females and >102 cm for males, obesity—body mass index ≥30 kg/m^2,^ low HDL ≤1.3 for females and 1.1 for males, hypercholesterolaemia—TC ≥ 5 mmol/l, hypertriglyceridaemia—TG ≥ 1.7 mmol/l, high TC/HDL-C >5, TG/HDL-C ≥ 1.49, high ApoB/ApoA >0.68

* ART information was available for 200 participants

*ART* antiretroviral therapy, *HIV* human immunodeficiency virus, *NVP* nevirapine, *EFV* efavirenz, *CVD* cardiovascular disease, *ApoB* apolipoprotein B, *Apo A* apolipoprotein A, *TC* total cholesterol, *HDL-C* high density lipoprotein cholesterol. *MET-min/wk* metabolic equivalent of task-minutes/week

### Predictors of metabolic CVD risk factors

People more than 50 years of age were more likely to be hypertensive (p < 0.05) and diabetic (p < 0.05) compared to people less 50 years of age. In addition an age of more than 50 years increased the likelihood of having metabolic syndrome (p < 0.05), a high concentration of TG (p < 0.05), a high TC/HDL-C ratio (p < 0.05) and a low concentration of HDL-C (p < 0.05) compared to an age of less than 50 years. Males were 2.94 times (p < 0.05) more likely to have a high TC/HDL-C ratio compared to females. People with a viral load of more than 50 copies/ml (>log 1.71) were less likely to be hypertensive but were more likely to have a low HDL-C concentration and a high ratio of ApoB/ApoA than people with a viral load of less than 50 copies/ml. People on ART for less than 60 months were less likely to have a high TC/HDL-C ratio than people on ART for more than 60 months. The likelihood of having a high TC concentration was 2.10 times (p < 0.05) and a high TG/HDL-C ratio was 2.98 times (p < 0.05) more in people with than in people without abdominal obesity. A low intake of fruit and vegetable was associated with a high concentration of TG (p < 0.05) (Table 2).

**Table 2:** Predictors of Metabolic CVD risk factors among ARV treated HIV infected people

Predictor variable	Metabolic CVD risk factors								
	HTN	Diabetes	Low HDL-C	High TC	High TG	High TC/ HDL-C	High Apo B/Apo A	High TG/HDL-C	Met S
Age (years)									
≤50	1 [reference]	1 [reference]	1 [reference]	–	1 [reference]	1 [reference]	1 [reference]	1[reference]	1 [reference]
>50	*4.67* ^*1*^ (1.94–11.2)	*5.66* ^*1*^ (1.36–23.5)	*2.27* ^*1*^(1.04–4.95)		*2.91* ^*1*^(1.12–7.55)	*3.29* ^*1*^ (1.35–8.01)	1.70 (0.90–3.21)	2.8 (0.84–5.68)	*1.10* ^*1*^ (1.02–1.18)
Gender									
Females	–	1 [reference]	–	–	–	1 [reference]	–	1 [reference]	–
Males		1.83 (0.46–7.20)				*2.94* ^*1*^ (1.13–7.65)		1.90 (0.21–17.6)	
Alcohol use									
No	1 [reference]	–	–	1 [reference]	–	–	–	–	–
Yes	0.57 (0.18–1.77)			1.69 (0.85–3.36)					
Tobacco use									
No	–	–	1 [reference]	–	–	–	–	–	1 [reference]
Yes			0.49 (0.19–1.23)						1.61 (0.30–8.70)
Log VL									
<1.71	1 [reference]	–	1 [reference]	–	–	–	1 [reference]	1 [reference]	–
>1.71	*0.08* ^*1*^ (0.01–0.63)		*3.82* ^*1*^ (1.51–9.63)				*3.83* ^*1*^ (1.73–8.46)	2.71 (0.84–8.75)	
ARV-duration									
>60 months	1 [reference]	–	–	–	1 [reference]	1 [reference]	–	1 [reference]	1 [reference]
30–60	2.32 (0.65–8.34)				0.48 (0.14–1.68)	*0.29* ^*1*^ (0.09–0.90)		0.49 (0.13–1.87)	0.48 (0.09–2.75)
<30 months	0.32 (0.66-8.19)				0.45 (0.14–1.46)	*0.31* ^*1*^ (0.11–0.89)		0.29 (0.07–1.23)	0.34 (0.05–2.60)
Abdominal obesity									
No	1 [reference]	–	1 [reference]	1 [reference]	1 [reference]	–	1 [reference]	1 [reference]	_
Yes	2.48 (0.69–8.89)		1.10 (0.26–4.66)	*2.10* ^*1*^ (1.09–4.05)	2.08 (0.75–5.82)		1.71 (0.88–3.35)	2.98^1^ (1.05–8.47)	
PA (MET-min/wk)									
>600	1 [reference]	–	1 [reference]	–	–	–	–	–	–
<600	0.27 (0.07–1.09)		0.54 (0.11–2.71)						
CD 4 count									
>500	–	–	–	–	–	–	–	–	1 [reference]
301–500									0.31 (0.05–1.96)
≤300									0.12 (0.01–1.29)
F/veg intake	–	–	0.92 (0.18–4.79)	–	0.56* (0.31–1.01)	–	–	0.67 (0.41–1.10)	–
Abd. obesity by PA	–	–	7.18 (0.74–69.7)	–	–	–	–	–	–
Gender by ARV months									
>60								1 [reference]	
30–60	–	–	–	–	–	–	–	0.46 (0.02–10.9)	–
<30								6.07 (0.45–28.8)	
Classification accuracy	81.7 %	95.3 %	67.8 %	69.6 %	84.1 %	87.4 %	61.8 %	82.6 %	91.0 %

Values are reported as adjusted odds ratio (confidence interval)

*PA* physical activity, *VL* viral load, *F/V* fruit and vegetable intake, *HDL-C* high density lipoprotein cholesterol, *LDL-C* low density lipoprotein cholesterol, *TC* total cholesterol, *TG* triglycerides, *Apo B* apolipoprotein B, *Apo A* apolipoprotein A, *Met S* metabolic syndrome, *HTN* hypertension, *ARV* antiretroviral, *MET-min/wk* metabolic equivalent of task-minute/week, *Abd* abdominal

^1 ^p-value < 0.05 is significant

* p = 0.05, marginally significant

### Framingham risk scores

None of the 164 participants according to the Framingham estimation had a high risk of developing a CVD event in the next 10 years. However, about 6.7 % had a moderate risk and majority had a low risk (93.3 %).

### DAD risk scores

Of the 164 participants, 68.9 % had a low risk, 27.4 % had a moderate risk and 3.7 % had a combined high and very high risk for developing a CVD event in next 5-years. However, considering all (214) participants, 66.8 % had a low risk, 29 % had a moderate risk, 1.9 % had a high risk and 2.3 % had a very high risk for developing CVD in next 5-years.

Comparison of the Framingham risk scores with the DAD risk scores in 164 participants who met the Framingham criteria gave a level of agreement of 73.8 % (Kappa = 0.23; 95 % CI 0.10–0.35; p value 0.001) (Table 3).

**Table 3:** Comparison between CVD risk estimation using DAD and Framingham equations

Cardiovascular disease risk	FRAMINGHAM (N = 164)			
	Low risk (<10 %)	Moderate risk (10–20 %)	High risk (>20 %)	
DAD (N = 164)				
Low risk (<10 %)	113	0	0	
Moderate risk (1–5 %)	37	8	0	
High risk and very high risk (>5 %)	3	3	0	
Agreement	73.8 %			
Kappa (p-value)	0.23 (0.001)			
95 % CI for Kappa	0.10–0.35			

	DAD risk estimations on all participants			
	Low risk (<1 %)	Moderate risk (1–5 %)	High risk (5.1–10 %)	Very high risk (>10 %)
All participants (N = 214)	143 (66.8 %)	62 (29 %)	4 (1.9 %)	5 (2.3 %)

*N* number, *DAD* data collection on adverse effects of anti-HIV drugs, *CVD* cardiovascular disease, *CI* confidence interval

## Discussion

Although our study population was young, using the DAD risk equation 31.1 % of participants had a 5-year moderate to high CVD risk. Several factors may play a role in causing this high risk. In our study, most people were physically active (75.8 %). However, the use of a different instrument for data collection may explain variations observed in physical activity between our study and others [5, 31]. Most participants in our study cited non availability of fruits and vegetables coupled with unaffordability as reasons for low intake. This low intake remains a major challenge as it increases the risk of nutritional deficiencies [32, 33] and CVD incidence and mortality [34]. Consistent with our findings, a low intake of fruit and vegetable was reported among the general population of Dikgale HDSS [35].

A high rate of unemployment was observed in this study, as was reported in the general population in the same community [35]. This high unemployment rate coupled with stigma related to HIV infection, may predispose the HIV infected people to high levels of stress. Given that chronic stress predicts the occurance of CVD [36], interventions aimed at creation of jobs among HIV infected people may therefore play an important role in indirectly reducing the risk for CVD, by alleviating stress associated with unemployment.

Among metabolic risk factors, hypertension was observed in nearly a quarter of participants, consistent with a study in Senegal [37]. However other studies that included younger populations reported lower prevalence rates [6, 38–40]. The prevalence of hypertension was similar between males and females. Of the 42 HIV infected participants with a history of hypertension and were on medication, approximately 60 % had their blood pressure controlled, while 40 % had a raised blood pressure despite being on medication. Factors contributing to the uncontrolled blood pressure include poor adherence to treatment, high salt intake and large alcohol consumption (>3 drinks per day) [41]. Older age was a predictor of hypertension, and people with a low viral load were more likely to be hypertensive. Diabetes mellitus (DM) in males and females was low as reported among people on ART in other African studies [5, 38, 42, 43]. Unlike our study findings, a high prevalence of diabetes mellitus was reported among HIV negative and ART naïve HIV positive people in Dikgle HDSS [22], probably due to an older age than that of our cohort. The overall prevalence of diabetes mellitus among South Africans aged 30 years and above is estimated at 9 % equaling a 9.3 % prevalence for United States of America [44]. South Africa is undergoing epidemiological transition and prevalence of diabetes mellitus is thus expected to rise in future [45]. The prevalence of obesity (10 %) and abdominal obesity (22 %) in our study differed from prevalences reported from Africa [6, 46] and Asia [40, 47]. According to Crum-Ciaflone et al. (2008) [48], the variations in obesity and abdominal obesity may partly be explained by differences in ART duration and the different cut-off values for the BMI and waist circumference used in various studies. Females in our study were more likely to be obese than males, although the difference was not significant due to the small number of participants [49]. The prevalence of metabolic syndrome was low and similar in males and females. Reported prevalences of metabolic syndrome vary, possibly resulting from its heterogeneous nature and variations in prevalence of its components [39, 50–52].

The prevalence of hypertriglyceridaemia was low, maybe due to the NNRTI based regimen used. The use of a stavudine containing regimen in certain other studies [40, 53, 54] may explain why a prevalence more than twice as high was observed in those studies. In our study high concentrations of TG, were more common in males than in females, but gender could not predict a high TG concentration in multivariate analysis. Abdominal obesity and older age were significant predictors of hypertriglyceridaemia as previously reported [55]. High TG levels in our study were associated with low intake of fruit and vegetables as has been reported in other studies [32, 33]. Although ART increases lipid levels, HDL-C may not return to normal levels thus high prevalence of low HDL-C has been observed in this and other studies [6, 38, 39]. Older age and high viral load were independent predictors of a low HDL-C concentration. These results show the importance of suppressing the viral load to minimize the risk for developing low HDL-C concentration when people on ART become older. Hypercholesterolaemia was present in a third of the participants which is similar to results from other studies [5, 6, 38, 39]. Visceral lipohypertrophy increased the likelihood of having a high total cholesterol concentration. None of our participants was using lipid lowering drugs, possibly accounting for the high proportions of dyslipidaemia. Lipid ratios are regarded as better predictors of CVD than individual lipids [56, 57]. A high ApoB/ApoA ratio was present in nearly 50 % of participants a possible reflection of high prevalence of low HDL-C. Its association with high viral load in our study predicts the benefits of effective suppression of virus load. Nearly 15 % of participants mostly males had a high TC/HDL-C ratio. Older age, longer ART duration and male gender, were predictors of a high TC/HDL-C ratio. These findings have important implications as these variables may increase the risk of developing CVD. A high TG/HDL-C ratio present in nearly a fifth of the participants mostly males was associated with abdominal obesity.

An earlier study conducted among ART naïve HIV infected and HIV negative people in Dikgale Health and Demographic Surveillance System site reported a high prevalence of CVD risk factors which was similar between the two groups [22], but a worse CVD risk profile compared to that of people on ART in the present study. Except for hypercholesterolaemia, the prevalence for most CVD risk factors in the present study is lower than that of ART naïve HIV infected and HIV negative in that study. A high prevalence of CVD risk factors among the general population in this rural area has previously been reported [58]. The fact that people on ART have monthly consultations at clinics and are informed of healthy lifestyles needed to manage co-morbidities could possibly explain the lower prevalence of CVD risk factors compared to ART naïve and HIV negative from same locality. Furthermore majority of participants (96 %) in the present study were receiving NNRTI based regimen associated with lesser CVD risk compared to PI based regimen [27].

Our study found a 73.8 % level of agreement between the Framingham and DAD risk estimation equations, which was similar to an agreement level of 77.4 % reported by Nery et al. (2013) [59]. Despite this level of agreement observed in our study, the Framingham equation underestimated the risk for CVD in 43 of the 164 participants, when compared to the DAD equation. These results suggest that the use of the Framingham equation in people infected with HIV receiving ART may lead to the exclusion of some individuals to benefit from more aggressive CVD prevention. The low rate of CVD risk as measured by the Framingham equation in our study may be that our cohort was relatively young and predominantly composed of females. Similar to our findings, literature suggests that the Framingham equation underestimates the risk of CVD in South Africans [60]. However, contrary to our findings, the Framingham equation overestimated the 10-year CVD risk among HIV infected Thais [9] and Brazilians [59] when compared to DAD equation.

Our study is one of the first that assessed a wide range of CVD risk factors as well as determined the 10-year CVD risk for persons with HIV infection on ART in a rural population in South Africa. Limitations of our study include its cross-sectional design, therefore we cannot conclude that the associations between covariates and CVD risk factors are causal. Information on tobacco use, alcohol use, physical activity and fruit and vegetable intake was obtained using the WHO STEP questionnaire. This is considered to be a reliable instrument, however recall bias may have influenced the results. Non-random sampling was used to recruit participants. However, recruitment was conducted for a whole month cycle, giving all patients collecting their medication equal opportunity to participant. Follow up on participants who failed to turn up for the agreed scheduled date helped to reduce selection bias. While our sample may not be representative of the whole population of HIV infected South Africans receiving ART our study provides valuable and useful information for comparison with other published studies from both developing and developed countries. We also acknowledge the small sample size of our study.

## Conclusion

The CVD risk factors were common among this rural population on ART. While 6.7 % of participants had a moderate risk, none had a high risk of developing a CVD event in the next 10 years, according to Framingham risk score equation. However, the high proportion of people with a moderate to very high risk of developing CVD in the next 5 years according to DAD risk score equation clearly represents a considerable health burden that can possibly be rectified by increasing educational programs on CVD prevention for people on ART. Whilst the DAD equation was not developed for Africans, its use instead of the currently used Framingham risk table in people on ART in South Africa, to identify people with a high CVD risk may help to reduce the burden of CVD on the health system. There is a need however to develop and evaluate a race/ethnicity-specific CVD risk estimation tool for HIV infected Africans.
